# A Retrospective Analysis Evaluating the Impact of Neighborhood Deprivation on Birth Weight in Phoenix, Arizona

**DOI:** 10.3390/ijerph22010112

**Published:** 2025-01-15

**Authors:** Kristin D. Mickelson, Megan Witsoe, Brittany Krzyzanowski, Pooja Doehrman, Samantha Dinh, Guangying Zhou, Jacqueline Nguyen

**Affiliations:** 1 School of Social & Behavioral Sciences, Arizona State University, 4701 W. Thunderbird Road, Glendale, AZ 85306, USA; 2 School of Medicine, Creighton University, 3100 N Central Ave, Phoenix, AZ 85012, USA; 3 Barrow Neurological Institute, 2910 N 3rd Ave, Phoenix, AZ 85013, USA; 4 St. Joseph’s Hospital and Medical Center, Dignity Health, 350 W Thomas Rd, Phoenix, AZ 85013, USA

**Keywords:** racial disparities, birth weight, low birth weight (LBW), area deprivation index (ADI), environmental discrimination, maternal health, healthcare disparities

## Abstract

Background: Health inequities begin before birth and are influenced by pregnancy conditions, race/ethnicity, social class, and environment. Research indicates that, in the United States, Black women are significantly more likely to have low-birth-weight babies compared to White women. Interestingly, Hispanic women in the United States do not experience this birth weight inequity. The reasons for this disparity remain unclear. Both Hispanic and Black women face discrimination, and this is often cited as a primary reason for the higher prevalence of low-birth-weight babies among Black women. One type of discrimination that is less examined is neighborhood deprivation. Method: This study systematically examined the impact of various sociodemographic and pregnancy predictors among 9607 women in Phoenix, Arizona. Using multilevel modeling, we analyzed whether neighborhood deprivation (using the Area Deprivation Index) influenced the association between demographic and pregnancy risk and protective factors on birth weight outcomes. Results: Consistent with prior research, we found that Black and Asian women had lower-birth-weight babies than White women, while Hispanic women did not show a significant difference from non-Hispanic women. Additionally, multilevel modeling suggested that increased neighborhood deprivation tends to exacerbate the impact of some risk factors (e.g., race) and reduce the impact of specific protective factors (e.g., gestational age) on birth weight. Conclusion: These findings suggest that both place and individual factors synergistically influence birth weight outcomes. Moreover, the results underscore the importance of targeting interventions to enhance resources among those who live in the most deprived neighborhoods.

## 1. Introduction

Birth weight is one of the most crucial indicators of neonatal health and has significant implications for long-term outcomes [1]. Low birth weight (LBW) is a major contributor to newborn mortality and is closely linked to increased youth mortality and a higher risk of chronic illnesses [1]. Maternal health conditions, such as hypertension, nutrition, and access to prenatal care, are known to contribute to LBW [2]. However, to effectively mitigate LBW, it is imperative for current research to identify physical, psychological, financial, and social influences on both the macro-level and individual levels that contribute to the aforementioned maternal health conditions. A comprehensive review of the literature shows that socioeconomic status, perinatal medical risks, and maternal lifestyle are the most predictive factors of LBW [3]. In our previous study, we conducted face-to-face interviews with a diverse sample of 329 pregnant women in Phoenix, including Black, Hispanic, and White participants, to systematically investigate the impact of various forms of discrimination—daily, environmental, and vicarious—on continuous birth weight [4]. We also explored the roles of familism, prayer, and discrimination attribution as potential buffering factors in this relationship. Our linear regression analyses revealed that prayer emerged as a significant resilience factor, particularly for Hispanic women, who appeared to experience protective benefits in the relationship between vicarious and daily discrimination and birth weight, especially when adjusted for gestational age. Notably, for the current study, we found that perceived environmental discrimination (e.g., pollution, crime, safety) was a critical factor associated with adverse birth weight outcomes, underscoring the complex interplay between discrimination and maternal health [4,5,6]. To further explore the relationship between discrimination and maternal health, it is important to understand whether, at the macro-level, environmental discrimination influences the association between risk and protective factors on the disparity in birth outcomes among Black, Hispanic, and White women in the United States [7].

The Area Deprivation Index (ADI), developed by Gopal Singh in 2003, is one tool that can be used to assess environmental and socioeconomic disadvantages by evaluating 17 census variables, including income, education, housing, and employment status [8]. The ADI helps pinpoint areas where residents face economic hardships and limited access to essential resources and services [8,9]. The ADI is considered to be the “most heavily independently validated, scientific tool for US neighborhood-level (exposome-level) disadvantage that exists today” [10]. Extensive research has demonstrated a negative association between ADI and birth weight, highlighting the significance of socioeconomic factors on neonatal health [9,11]. An ADI analysis in another U.S. state identified possible vicinity-specific factors, such as close proximity of an academic health institution, on birth outcomes [12]. However, it is unclear whether neighborhood deprivation differentially impacts macro-level (i.e., societal) factors, such as environmental conditions, and micro-level (i.e., individual) factors, such as sociodemographics and pregnancy variables, thereby affecting birth weight outcomes.

In Phoenix, Arizona, several organizations, including Women, Infants, and Children (WIC), the Dignity Health MomMobile mobile prenatal care unit, and Native Health Start, serve as community interventions to address health resource gaps. While these organizations represent initial advancements toward equality, it is vital that resources are distributed with equity, rather than equality, in order to mitigate healthcare disparities [13,14]. Additionally, the presence or absence of other community facets, such as churches, schools, and community leaders, may further contribute to these disparities [14], and maternal proximities to these resources have yet to be investigated in Phoenix, Arizona. This research aims to evaluate the relationship between neighborhood deprivation (as assessed with the ADI) and birth weight in one large metropolitan city in the United States with a diverse population. This research specifically focuses on determining environmental factors within the vicinity of maternal residential areas that could work to exacerbate or buffer against the disparities in healthcare to guide program development and interventions. A secondary goal is to determine whether the previous findings regarding perceived environmental discrimination and birth weight replicate with environmental discrimination as assessed via neighborhood deprivations.

## 2. Methods

This retrospective cohort study was approved by the St. Joseph Hospital and Medical Center Institutional Review Board (IRB # PHX-24-500-032-71-47).

A retrospective cohort study of pregnant mothers who delivered from October 2018 to December 2020 at St. Joseph’s Hospital was performed. To ensure consistency in our investigation, we maintained the same time frame as used in the previous study [4]. We used macro-level data with a representative sample of the population of Phoenix to evaluate the environmental factors influencing discrimination and birth outcomes. The total number of live births in Phoenix 2019 and 2020 was 50,998 and 49,191, respectively. Based on the trends for the Phoenix metropolitan area, we estimate the total number of births during our prior study enrollment period was approximately 100,000. We calculate that a representative sample would have a minimum of 5% of all live births in the study period. We were able to obtain a sample of 12,001 from a retrospective chart review of data from St. Joseph’s Hospital and Medical Center (SJHMC) from 2018 to 2020. SJHMC was the first hospital in Phoenix, Arizona, and is a highly respected and large non-profit medical institution. SJHMC accepts insured and uninsured patients and focuses on health equity. Thus, we believe that the sample was fairly representative of the larger Phoenix population of pregnant women. After deleting birth weight (grams) data that were recorded as zero due to miscarriage/terminated pregnancy or patients having their babies at another hospital (*n* = 2340), less than 400 g (*n* = 50), missing (*n* = 3), or over 5500 g (*n* = 1), the final sample size for analyses was 9607 pregnant women. There were no sampling biases or inclusion/exclusion criteria with respect to sociodemographics (e.g., race/ethnicity, age, etc.); all medical records for women giving birth at this hospital were pulled from the stated time period.

Neighborhood deprivation was assessed using the Area Deprivation Index (ADI) [15]. The ADI for this sample was calculated using participant 5-digit zip codes. Zip code level data for the ADI in 2020 were obtained from the University of Wisconsin Madison [8,16]. Using geographic information systems (GISs), each patient was geocoded to their home address of residence. We achieved a 98.83% match rate for the full sample wherein 11,861 patients were assigned to the latitude and longitude coordinates of their home address. After geocoding, these patients were assigned to an ADI decile based on the ADI value of the zip code that contained the latitude and longitude of their home address. The ADI was coded such that a low value indicates less deprivation and a higher value indicates more deprivation.

With respect to pregnancy conditions, we created two separate risk scores. One risk score consisted of eclampsia, pre-eclampsia, and gestational hypertension (also known as risk3 = scores ranged from 0 to 3). In other words, those with a risk3 score of 3 had all three of the conditions (eclampsia, pre-eclampsia, and gestational hypertension); a score of 2 means they had two of the three conditions and a score of 1 means they only had one of the three conditions. The other risk score consisted of obesity and gestational diabetes (also known as risk2 = scores ranged from 0 to 2) and was scored similarly to the other measure, with a score of 2 meaning a woman had both obesity and gestational diabetes and a score of 1 meaning that she only had one of these two conditions.

Because the women in this sample were nested within zip codes, we used multilevel modeling to assess their within- and between-zip code associations in regard to birth weight. Multilevel modeling (MLM) does not require the independence of observation assumption; instead, it allows for the assessment of the group effects on the individual, as well as the analysis of within- and between-level variance. In this particular case, zip codes were clustered by ADI codes for the degree of neighborhood deprivation. All assumptions for MLM were met and the sample size was adequate with 171 Level-2 groups (i.e., zip code) with three or more participants in each zip code [17]. A minimum of three participants per zip code is required for nested analyses, creating the final sample size for the multilevel modeling of 4847 participants nested within 171 zip codes.

For Level-1, we examined the individual micro-level predictors (sociodemographics and pregnancy variables). Continuous Level-1 predictors (i.e., age, gravidity, parity, gestational age) were centered around the grand sample mean to assess the deviation from the sample mean score on a given predictor. The sole Level-2 predictor of neighborhood deprivation (ADI) was re-coded to 0 to 9 to allow for it to be examined without centering in order to assess the pure association of the ADI on the individual-level slopes. The random components from the Level-2 slope models were removed because of the complexity of the model and because we can assume that the error is minimal given that the Level-1 predictors are based on medical records. Because the effects are treated as fixed rather than random, we can only generalize our results to those sampled.

## 3. Results

Descriptive statistics of the sample reveal that the average birth weight for this sample was 3249.60 g (SD = 583.43, Range = 405–5455). Additionally, as shown in Table 1, and as consistent with prior research [18], Black and Asian women had babies with significantly lower birth weights than White and Other Race (American Indian/Alaska Native, Native Hawaiian/Pacific Islander, Multiracial) women (F(3, 9457) = 21.57; *p* < 0.001). Additionally, and also consistent with prior research [19], Hispanic women had babies with significantly higher birth weights than non-Hispanic women (F(1, 9303) = 18.64; *p* < 0.001).

An examination of low birth weight (i.e., 2500 g) percentages showed that 7.70% of the sample had a baby classified as low birth weight. Race/ethnic breakdowns matched the results for absolute birth weight. Specifically, Black (11.42%) and Asian (10.63%) women were significantly more likely to have a low-birth-weight baby than White (7.07%) and Other Race (6.20%) women (χ^2^(3) = 31.08; *p* < 0.001)). With respect to Hispanic ethnicity, 9.3% of non-Hispanic women had a low-birth-weight baby compared to only 6.4% of Hispanic women (χ^2^(1) = 26.71; *p* < 0.001), which supports the well-established Latina Perinatal Paradox [19].

We also examined the association between age and marital status on birth weight and found that older age was correlated with higher birth weights and that married/cohabiting women had significantly higher-birth-weight babies than single women (there was no difference in birth weight for separated/divorced/widowed women). Analyses of various pregnancy variables (i.e., parity, gravidity, baby sex, and gestational age) found that gravidity, parity, and gestational age were all positively correlated with greater birth weights. All of these associations between sociodemographics and pregnancy variables with birth weight are in line with prior research [3]. Interestingly, the risk scores had opposing correlations with birth weight. Specifically, a higher score on risk3 (eclampsia, pre-eclampsia, hypertension) was correlated with greater birth weights. This result is contrary to the current literature on pregnancy factors and birth weight; we are unclear if this is an artifact of this sample or some cumulative buffering influence on birth weight. Future research needs to determine if this correlation replicates in other samples. On the other hand, a higher score on risk2 (obesity, gestational diabetes) was correlated with lower birth weights. The prior literature has found inconsistent associations with maternal obesity and diabetes on birth weight, with some finding a positive association and others a negative association, as we found [20]. Finally, as commonly seen in the literature [21], female babies had significantly lower birth weights than male babies. See Table 2 for the bivariate correlations and means for categorical variables.

As stated above, we used the five-digit zip code for women in the sample to calculate their Area Deprivation Index (higher scores represent greater neighborhood deprivation). For the final sample for the multilevel modeling analyses (*n* = 4847), the ADI for this sample ranged from 1 to 10, with a mean of 5.98 (SD = 3.31), suggesting that the neighborhoods of this sample were moderately deprived on average.

Prior to the main analyses, we tested the unconditional model, which was significant, suggesting significant within zip code variations in regard to continuous birth weight. Given the large number of analyses, we will summarize the results in narrative form only; however, all of the coefficients, intercepts, and significance levels are presented in Table 3 and Table 4. The first set of analyses consisted of a conditional Level-1 and unconditional Level-2 model. Using a model-building approach, we first entered the pregnancy variables (parity, gravidity, baby sex, gestational age, risk2, and risk3) simultaneously into the Level-1 model with birth weight as the outcome. Then, we removed any non-significant predictors (i.e., gravidity) before adding the sociodemographic variables (age, marital status, race, Hispanic ethnicity). Finally, we removed the non-significant sociodemographic predictors (i.e., marital status, Hispanic ethnicity) to examine our final Level-1 model.

As shown in Table 3, and in line with the bivariate correlations, higher birth weights were predicted by older age, greater parity, longer gestation, and a higher score on risk3 (eclampsia, pre-eclampsia, gestational hypertension). On the other hand, Black and Asian women had significantly lower-weight babies compared to White women. Additionally, having a female baby and a higher score on risk2 (obesity, gestational diabetes) predicted significantly lower birth weights.

To examine cross-level interactions by neighborhood deprivation, we added the ADI as the Level-2 predictor of the slopes and intercept, leaving the intercept random and fixing the slopes. Additionally, by re-coding the ADI to range from 0 to 9, we left the predictor uncentered, meaning that the intercept is the average birth weight at the lowest level of deprivation (i.e., in the wealthiest neighborhoods). As shown in Table 4, neighborhood deprivation interacted to impact the intercept such that, as neighborhood deprivation increases, birth weight decreases. This finding is consistent with other research in the United States examining neighborhood deprivation using other measures besides the ADI [22]. Additionally, neighborhood deprivation impacted several of the slopes. Neighborhood deprivation exacerbated the association between race and birth weight; specifically, living in more deprived neighborhoods increases the racial disparity in birth weight for Black women (but not Asian women). Women of other races did not differ significantly in birth weight relative to White women at the main effect level; however, neighborhood deprivation significantly reduced birth weights for women of Other races. Prior research examining the interaction between neighborhood deprivation, race/ethnicity, and birth weight has found inconsistent results; whereas some find a stronger link between deprivation and lower birth weights for Black women, others either find this exacerbation for non-Hispanic White women or no race/ethnic differences [22]. With respect to protective factors such as parity and gestational age, neighborhood deprivation weakens those protections. In addition, the baby sex gap in birth weight is reduced by 12.72 g for every unit increase in neighborhood deprivation. The weight difference between male and female babies decreases from 207.90 g to only 93.42 g for those living in the most deprived neighborhoods. While other research has examined neighborhood deprivation through multilevel modeling [23], to our knowledge, we are one of the first to use the ADI measure in multilevel modeling to examine the effects of neighborhood deprivation on birth weight. Moreover, among prior studies conducted in the United States, none have been performed in the Southwestern region, which is distinct for its large Hispanic and migrant populations.

Finally, we examined whether neighborhood deprivation predicted low birth weight (less than 2500 g) using multilevel modeling. As with continuous birth weight, prior to the main analyses, we tested the unconditional model, which was significant, suggesting significant within zip code variation in regard to low birth weight. Given the large number of analyses, we will be summarizing the results in narrative form only; however, all of the intercepts and odds ratios are presented in Table 5.

The first set of analyses consisted of a conditional Level-1 and unconditional Level-2 model. Using a model-building approach, we first entered the pregnancy variables (parity, gravidity, baby sex, gestational age, risk2, and risk3) simultaneously into the Level-1 model, with low birth weight acting as the dichotomous outcome. Then, we removed any non-significant predictors (i.e., gravidity, baby sex) before adding the sociodemographic variables (age, marital status, race, Hispanic ethnicity). Finally, we removed the non-significant sociodemographic predictors (i.e., age, Hispanic ethnicity) to examine our final Level-1 model.

As shown in Table 5, women with greater parity, longer gestation, and a higher score on risk3 (eclampsia, pre-eclampsia, gestational hypertension) had lower odds of having a low-birth-weight baby. On the other hand, Black women had significantly higher odds of having a low-birth-weight baby compared to White women. Additionally, women who were married/cohabiting and had a higher score on risk2 (obesity, gestational diabetes) had higher odds of having a low-birth-weight baby.

To examine cross-level interactions by neighborhood deprivation, we added the ADI as the Level-2 predictor of the slopes and intercept, leaving the intercept random and fixing the slopes. Additionally, as before, we used the re-coded ADI (range = 0 to 9) and left the predictor uncentered, meaning that the intercept is the average odds of having a low-birth-weight baby at the lowest level of deprivation (i.e., in the wealthiest neighborhoods). The ADI was not significant for the intercept or any of the slopes; thus, neighborhood deprivation did not interact with the Level-1 predictors to influence the odds of having a low-birth-weight baby.

## 4. Discussion

The national racial disparities in birth weight were reflected in our diverse Southwest U.S. metropolitan population, with a significant proportion of Black women having lower-birth-weight babies than White women [24,25]. On the other hand, as supported by prior research [26], Hispanic women were less likely to have lower-birth-weight babies relative to non-Hispanic women (even in similarly deprived neighborhoods). This paradox of Hispanic women, who face similar disparities regarding access to care, socioeconomic, education, and language barriers to Black women yet have higher-birth-weight infants, highlights the need to identify factors that negatively and positively impact maternal and child health. Other studies evaluating racial and ethnic disparities in obstetric outcomes often examine the impact of race through the lens of immigration by focusing on the country of origin [27]. The immigrant paradox, defined as first-generation immigrants experiencing poorer health outcomes despite starting pregnancies in better health, may help explain why women from different countries of origin face disproportionate obstetrical risks [28]. This introduces contradictory findings in comparison to our own, as we observe that Hispanic women in the U.S. tend to experience better birth weight outcomes despite similar socioeconomic disadvantages. However, the immigrant paradox and the effects of immigration stress offer important contexts for these disparities [28], adding complexity to our study by providing another theory regarding racial differences and birth weight and showing that factors beyond race, such as immigration status and cultural resilience, must be considered when analyzing maternal health disparities.

In order to make a meaningful impact in regard to addressing health equity across racial and ethnic lines, we must better understand the influencing factors and opportunities for building resilience in vulnerable communities [29,30]. The Area Deprivation Index (ADI) is one proxy for environmental discrimination, helping us understand how factors such as income, education, housing, and employment status affect these communities. However, we still have a limited understanding of how these socioeconomic indicators directly relate to the physical environments of pregnant mothers and interact with other environmental stressors (e.g., climate, crime, and pollution).

The ADI provides a mechanism to assess how social and economic factors in a specific environment contribute to racial disparities in birth weight. Our results support the notion that neighborhood deprivation exacerbates the impact of risk factors and negates the impact of protective factors, such as greater parity or gestational age, on birth weight [31]. Our study also supported the concept that neighborhood deprivation weakens the link between a baby’s sex and birth weight due to the reduction in the baby sex gap in birth weight as neighborhood deprivation levels increase. Furthermore, the relationship between individual patients’ perceptions of environmental discrimination and lower-birth-weight babies [4] was also mirrored at the macro-level when using the ADI as a measurement of environmental discrimination.

One seminal result in terms of neighborhood deprivation found that the negative impact of the ADI weighed more heavily on Black mothers in our population, highlighting vulnerabilities in this racial group [32]. Black mothers in neighborhoods with greater deprivation experienced even greater disparities in birth weight compared to White women. This increased risk of neighborhood deprivation for Black mothers may be interpreted as an example of weathering [33]—the accumulation of racial stress over Black women’s lives contributes to the observed pattern of racial disparities in maternal health and birth outcomes that increase with maternal age. Perhaps this theory also explains why our study found that Black women in areas with a high ADI were not protected by gestational age, parity, or the baby’s sex. Similar findings of racial discrepancies in obstetric outcomes, such as non-invasive prenatal testing adherence, have been reported [34]. Findings reveal that Black women have lower adherence rates to prenatal tests, furthering healthcare disparities [34]. These similar findings continue to demonstrate that systemic barriers and healthcare access issues disproportionately affect Black women, emphasizing the effect of neighborhood deprivation.

Insight into racial disparities in pregnancy outcomes in the U.S. can be elucidated by comparing results from different healthcare systems. A Swedish study found that mothers residing in socially deprived areas were more likely to have severely low-birth-weight neonates, stillbirths, and small-gestational-age neonates [35]. However, when accounting for social and individual maternal factors, only the increased likelihood of small-gestational-age neonates was significant in severely deprived areas [35]. Thus, mothers living in deprived areas had a lower risk of preterm births than what was expected, which may be related to the free antenatal and obstetric care that is provided and the ethnic homogeneity of Sweden in contrast to the U.S. [35,36]. In New Zealand, higher deprivation indices were associated with adverse birth outcomes for minority Maori and Asian women, specifically in terms of racism, poverty, and barriers to accessing care [37], which are similar factors influencing perinatal outcomes in the U.S. Comparisons between countries are limited due to varying factors that are accounted for in each deprivation index. Although zip code and neighborhood deprivation indices have been extensively studied in developed nations [36], the majority of birth weight and perinatal outcome studies reported on developing countries assess only national results [38]. Assessing our results in an international context reiterates how the interactions of different healthcare systems with the population produce varying birth weights.

To create health equity in marginalized communities, interventions must focus on improving access to prenatal care and addressing environmental stressors. Expanding access to affordable healthcare through telemedicine and mobile clinics can help overcome barriers like transportation [39]. Culturally tailored health education can bridge gaps in care and trust, particularly for women facing language or cultural barriers [40]. Addressing social determinants of health, such as economic insecurity and access to food, through job training and financial aid programs can improve barriers to care [41]. Additionally, implicit bias training for healthcare providers and ensuring a diverse healthcare workforce promotes equitable treatment [42]. Resources should be allocated more equitably in neighborhoods with high socioeconomic deprivation and limited access to care, being more heavily concentrated in these areas as these communities face compounded stressors that others may not [43]. These interventions collectively can reduce disparities and promote a healthier, more equitable future for marginalized communities.

While this study is the first to our knowledge to examine the role of neighborhood deprivation (using the ADI) on birth weight outcomes through multilevel modeling among a large sample of racially diverse women in the United States, there are some limitations. First, we were limited in terms of the sociodemographic variables that were collected, and no psychosocial constructs were assessed to allow for a replication with our previous study. Second, there were considerable data entry errors and omissions in the medical records, impeding our ability to assess pre-pregnancy BMIs and substance use/abuse levels, both of which have been found to be significant predictors of birth weight [3]. Third, because of inadequate sample sizes within the zip code nesting, our final sample was roughly one-third the size of the original sample. Fourth, as the data were only collected from Arizona, the results may be specific to this area. Lastly, we are unable to explain the unusual finding of eclampsia/pre-eclampsia/hypertension being related to higher birth weights. We are perplexed by this result. We tested to see if it was due to a suppression effect, and it was not; it consistently appeared even when we looked at each condition separately. We suggest that further research and replication is needed on other samples to determine if it is an artifact or something specific to this sample.

Future studies need to replicate our findings in other areas of the United States to discern the impact of the ADI on birth weight with multilevel modeling. Additionally, research needs to include psychosocial variables, such as perceived discrimination and resilience, to ascertain whether neighborhood deprivation has a similar exacerbating and negating effect on these factors, respectively. Finally, it would be beneficial to conduct qualitative studies to gain insights into individual and community perceptions regarding neighborhood deprivation to help target the most effective interventions.

## 5. Conclusions

Health disparities in birth outcomes among Black, Hispanic, and White women in the United States have continued to persist despite community interventions aimed at addressing these inequalities. While these interventions are a step toward equity, neighborhood deprivation continues to prevent equitable access to resources, which contributes to adverse birth outcomes in Black populations. The ADI provides an additional method for analyzing the impact of neighborhood deprivation on birth weight by utilizing census variables to determine a neighborhood’s socioeconomic circumstances. The current analysis demonstrated that neighborhood deprivation interacts with person-level factors to influence birth weight, suggesting that women living in neighborhoods with fewer resources are at increased risk of having lower-birth-weight infants than their more affluent counterparts. More importantly, protective factors, such as parity and gestational age, are attenuated in their effects on birth weight for those living in more deprived neighborhoods. These data continue to support the relationship between poor socioeconomic status and birth weight, as well as the impact of an individual’s perceived environmental discrimination on health outcomes. By identifying alleviating and aggravating factors in the environment, such as pollution, crime, and lack of access to resources, community interventions can be better designed to strategically target areas of deprivation to improve birth weight outcomes in minority communities.

## Figures and Tables

**Table 1:** Race/ethnic differences in birth weight.

	Birth Weight (g)		Low Birth Weight
	M	(SE)	%
Total Sample (*n* = 9607)	3249.60	(583.43)	7.70%
Race			
Asian (*n* = 301)	3120.80_a_	(556.66)	10.63%_ab_
Black (*n* = 1121)	3141.91_b_	(632.73)	11.42%_a_
White (*n* = 7636)	3268.33_b_	(574.39)	7.07%_b_
Other (*n* = 403)	3303.39_a_	(574.86)	6.20%_b_
Hispanic Ethnicity			
Hispanic (*n* = 5205)	3273.55_a_	(564.31)	9.27%_a_
Non-Hispanic (*n* = 4100)	3229.92_b_	(607.78)	6.40_b_

Note: Different subscripts in a column indicate that the means are significantly different at *p* < 0.05.

**Table 2:** Bivariate correlations and mean differences with birth weight.

	Birth Weight (g)	
	r	
Age	0.03	*
Gravidity	0.03	*
Parity	0.04	**
Gestational Age (days)	0.68	***
Risk3	0.07	***
Risk2	−0.11	***
Marital Status	M	(SE)
Married/Cohabiting (*n* = 4038)	3274.68_a_	(584.24)
Single/Never Married (*n* = 5381)	3231.02_ab_	(581.35)
Separated/Divorced/Widowed (*n* = 138)	3217.83_b_	(607.53)
Baby Sex		
Female (*n* = 4673)	3204.11_b_	(544.37)
Male (*n* = 4689)	3297.75_a_	(614.97)

Note: Different subscripts in a column indicate that means are significantly different at *p* < 0.05. * *p* < 0.05; ** *p* < 0.01; *** *p* < 0.001.

**Table 3:** Fixed Effect Level-1 multilevel modeling results for birth weight.

		Birth Weight (g)	
	Estimate		SE
Model Intercept	3296.37		(9.66)
Pregnancy Factors			
Parity	18.57	***	(3.29)
Baby Sex	−123.80	***	(13.26)
GA (days)	27.48	***	(0.41)
Risk3	100.12	***	(11.15)
Risk2	−55.55	***	(14.02)
Sociodemographics			
Age	4.63	***	(1.01)
Race			
Black	−118.38	***	(19.28)
Asian	−124.23	***	(33.07)
Other	10.52		(38.93)

Note: Parity, GA (gestational age), risk3, risk2, and age were all grand-mean centered. Baby sex: 0 = male, 1 = female. Race: White is the reference group. Risk3 = sum score of eclampsia, pre-eclampsia, and gestational hypertension. Risk2 = sum score of obesity and gestational diabetes. *** p < 0.001

**Table 4:** Level-2 Multilevel modeling results for birth weight.

					Birth Weight (g)	
				Estimate		SE
Model Intercept				3347.39		(24.40)
		ADI		−7.57	*	(3.36)
Pregnancy Factors						
		Parity—Intercept		37.77		(8.37)
		ADI		−2.80	**	(1.07)
		Baby Sex—Intercept		−207.90		(27.16)
		ADI		0.17	***	(3.91)
		GA (days)—Intercept		30.25		(0.88)
		ADI		−0.39	**	(0.12)
		Risk3—Intercept		54.79		(29.02)
		ADI		6.75		(4.12)
		Risk2—Intercept		−69.14		(38.17)
		ADI		0.02		(5.24)
Sociodemographics						
		Age—Intercept		5.95		(2.27)
		ADI		−0.19		(0.33)
		Race				
		Black—Intercept		−23.71		(42.04)
		ADI		−14.02	*	(6.18)
		Asian—Intercept		−82.61		(87.48)
		ADI		−6.28		(11.11)
		Other—Intercept		190.66		(77.13)
		ADI		26.24	*	(10.90)

Note: Parity, GA (gestational age, risk3, risk2, and age were all grand-mean centered. The intercept variance was left random and all slope variances were fixed. The intercepts are conditional, so only the significance of the ADI is reported. The ADI is coded such that a higher score indicates greater neighborhood deprivation. Baby sex: 0 = male, 1 = female. Race: White is the reference group. Risk3 = sum score of eclampsia, pre-eclampsia, and gestational hypertension. Risk2 = sum score of obesity and gestational diabetes. * *p* < 0.05; ** p < 0.01; *** p < 0.001.

**Table 5:** Fixed Effect Level-1 multilevel modeling results for low birth weight.

				LBW (<2500 g)		
			OR		CI	
Model Intercept			0.02	***	(0.01, 0.02)	
Pregnancy Factors						
	Parity		0.91	*	(0.83, 0.99)	
	GA (days)		0.86	***	(0.85, 0.87)	
	Risk3		0.68	***	(0.54, 0.85)	
	Risk2		1.56	***	(1.23,1.98)	
Sociodemographics						
	Marital Status		1.30	*	(1.01,1.67)	
	Race					
	Black		2.26	***	(1.47,3.50)	
	Asian		1.40		(0.65,3.02)	
	Other		0.74		(0.28, 1.96)	

Note: Parity, GA (gestational age), risk3, risk2, and age were all grand-mean centered. Marital Status: 0 = single, 1 = married/cohabiting (separated/divorced/widowed were not included because of the small number of participants in this status). Race: White is the reference group. Risk3 = sum score of eclampsia, pre-eclampsia, and gestational hypertension. Risk2 = sum score of obesity and gestational diabetes. * *p* < 0.05; *** p < 0.001.

## Data Availability

The data presented in this study are available on request from the corresponding author. The data are not publicly available due to it being connected with patient medical records.
